# Ileosigmoid knot strangles the sigmoid

**DOI:** 10.4103/0974-2700.55351

**Published:** 2009

**Authors:** Fatima Ezzahra Zahid, Karim Ibn Majdoub, Jihane Lamrani, Khalid Mazaz

**Affiliations:** Department of General Surgery, Universitet Hospital Hassan II, Fes, Morocco

Sir,

Ileosigmoid knot is an unusual clinical entity of small bowel obstruction in which the ileum wraps around the base of the sigmoid colon and forms a pseudo knot. We present a case of a 65-year-old man in whom the diagnosis of ileosigmoid knotting was made via emergency surgery.

Our patient was a 65-year-old man who arrived at the Emergency Department and was admitted with profound shock. He was complaining of acute worsening abdominal pain, vomiting and constipation for 2 days. On admission, the patient was febrile at 38°C, with a pulse rate of 50/min and blood pressure of 70/50 mmHg. Abdominal examination revealed a mildly distended abdomen with diffuse tenderness and positive rebound tenderness. No abdominal mass was palpable. His rectal examination was negative for blood. We treated the patient with intravenous normal saline and ordered laboratory work-up for chemistry, coagulation profile and Hem studies.

Abnormal laboratory parameters were mild anemia (Hb: 11g/dl), with leucocytosis (white cell blood count 26000/mm^3^) and blood urea of 0.8mg/ml. An abdominal X-ray showed small bowel air-fluid levels. A bedside sonography revealed free peritoneal fluid in the perihepatic and perisplenic spaces and between the distended intestinal loops. Because of the patient's instable status, a computerized tomography (CT) scan was deferred and the patient was taken to the operating room for an urgent laparotomy.

Intraoperatively, 2 L of blood-stained fluid was noted and Ileosigmoid knot (ISK) was observed. A sigmoid loop was found tightly encircling the congested and dilated loops of the ileum forming a knot. The sigmoid was not necrotic and was not resected because of the condition of the patient. Three meters of the distal ileum was frankly necrotic. No perforation was visualized. The 3 m of distal ileum was resected with cecum. A termino-lateral ileo-colic anastomosis was performed because of the shortness of the small intestine. We planned an elective sigmoidectomy at a later date.

Ileosigmoid knot is an uncommon lesion; however, it is a well-recognized condition in certain African, Asian and Middle Eastern nations. It was first reported by Parker in 1845. Its incidence in the general population is not known, but it is common in adult males and the peak incidence is in the fourth decade.[1]

Although the basic requirements for an ileosigmoid knot are still controversial, most radiologists agree that they include a hypermobile small intestine with markedly elongated mesentery and a redundant omega-shaped sigmoid colon with a long mesocolon and a short attachment at the base of the mesentery. Alver performed a new classification for ISK. Type I: The ileum (active component) wraps itself around the sigmoid colon (passive component) in a clockwise or counterclockwise direction. Type II: The sigmoid colon (active component) wraps itself around a loop of the ileum (passive component) in a clockwise or counterclockwise direction. Type III: The ileocecal segment (active component) wraps itself around the sigmoid colon (passive component). In most cases, the ileum (active component) wraps around the sigmoid colon rather than the reverse. In our case, we noted an omega-shaped sigmoid encircling the small intestine, corresponding to Type II Alver's classification.

The clinical features of ileosigmoid knot present with acute severe abdominal pain due to ischemia of the bowel associated with vomiting and abdominal distention and the in case of any delay in diagnosis, the general condition of the patient will deteriorate rapidly.[2]

Characteristic radiological features including a double closed loop obstruction, with the small intestinal loops in the upper left quadrant and the sigmoid loop in the right,[3] are often misdiagnosed preoperatively. Abdominal tomodensitometry also exhibits characteristic findings that may reveal a sigmoid volvulus, including characteristic whirlpool signs created by the twisted intestine and mesocolon, both created by the twisted intestine and mesentery and medial deviation of the descending colon with a beak-like appearance on its medial border.[3] CT can also reveal signs of bowel ischemia caused by strangulation, such as pneumatosis. Despite reports that describe the radiographic appearance and clinical features of the ileosigmoid knot, diagnosis of an ileosigmoid knot is difficult because of its infrequency and atypical radiographic findings. Consequently, this rare abdominal condition is usually diagnosed during exploratory laparotomy[3] and preoperative diagnosis is made in less than 20% of the cases. Even during surgery, ISK can be misdiagnosed because the macroscopic features of this unusual condition are not familiar to surgeons.[3]

The mortality rate is high, 25-47%,[3] mainly due to toxic shock from bowel gangrene. Prompt laparotomy and resection of the nonviable or doubtful intestines in a patient with antibiotic therapy and adequate fluid resuscitation are key factors to diminish the mortality rate. Some authors recommend performing sigmoid resection even if it appears to be macroscopically viable,[3] whereas in some reported cases the sigmoid is not resected.[3] Anastomosis of the small bowel and a Mikulicz-type exteriorization of the gangrenous sigmoid are recommended.[4] However, Hartmanns procedure is recommended when the gangrene has extended down to the upper rectum. In our case, the clearly gangrenous ileum was resected, the sigmoid was viable and was respected because of hemodynamic instability of the patient. The plan is for the patient to return for sigmoidectomy.

We conclude that the ileosigmoid knot is an unusual clinical entity of small bowel obstruction. It is a serious condition that rapidly progress to gangrene. Thus, early diagnosis and prompt treatment are essential despite difficulty in making a preoperative diagnosis.

## References

[1] Hirano Y, Hara T, Horichi Y, Nozawa H, Nakada K, Oyama K (2005). Ileosigmoid knot: Case report and CT findings. Abdom Imaging.

[2] Agaoglu N (2003). A rare cause of intestinal obstruction: Ileosigmoid knot. East J Med.

[3] Fouquet V, Berrebi D, De Lagausie P, Azeinfish S, Chalard F (2006). Ileosigmoid knotting in a child: The first case report in a French girl. Gastroenterol Clin Biol.

[4] Bawa D, Ikenna EC, Ugwu BT (2008). Ileosigmoid knotting: A case for primary anastomosis. Niger J Med.

